# Computational Methodologies for the *in vitro* and *in situ* Quantification of Neutrophil Extracellular Traps

**DOI:** 10.3389/fimmu.2019.01562

**Published:** 2019-07-10

**Authors:** Shane V. van Breda, Lenka Vokalova, Claire Neugebauer, Simona W. Rossi, Sinuhe Hahn, Paul Hasler

**Affiliations:** ^1^Laboratory for Prenatal Medicine, Department of Biomedicine, University Hospital Basel, Basel, Switzerland; ^2^Division of Rheumatology, Kantonsspital Aarau, Aarau, Switzerland

**Keywords:** neutrophil extracellular traps, myeloperoxidase, neutrophil elastase, citrullinated histone, machine-learning

## Abstract

Neutrophil extracellular traps (NETs) are a neutrophil defensive mechanism where chromatin is expelled together with antimicrobial proteins in response to a number of stimuli. Even though beneficial in many cases, their dysfunction has been implicated in many diseases, such as rheumatoid arthritis and cancer. Accurate quantification of NETs is of utmost importance for correctly studying their role in various diseases, especially when considering them as therapeutic targets. Unfortunately, NET quantification has a number of limitations. However, recent developments in computational methodologies for quantifying NETs have vastly improved the ability to study NETs. Methods range from using ImageJ to user friendly applications and to more sophisticated machine-learning approaches. These various methods are reviewed and discussed in this review.

## Introduction

Publications describing the formation of neutrophil extracellular traps (NET) have increased exponentially since their initial description in 2004 (1). Formed as a response of neutrophils to microorganisms and a host of other stimuli, NETs consist of decondensed chromatin released from the nucleus through the cytoplasm into the extracellular space (1). Nuclear and cytoplasmic components are mingled in the NETs and include antimicrobial peptides, such as myeloperoxidase (MPO), neutrophil elastase (NE) and, in certain instances, citrullinated histones (H3Cit) (1–3). NETs are believed to prevent dissemination and propagation of various pathogens (4–6). However, even though NETs might be beneficial, inappropriate function and tissue damage have been implicated in multiple pathologies i.e., pre-eclampsia (2, 7), diabetes and gestational diabetes (8–11), rheumatoid arthritis (RA) (3, 12, 13), systemic lupus erythematous (SLE) (14), community acquired pneumonia (15), sepsis (16), thrombosis (17), acute respiratory distress syndrome (18), and cancer (19, 20).

Clearly, it is evident that NETs are of considerable importance when studying innate immunity, understanding disease mechanisms or when using them as biomarkers or therapeutic targets. Thus, accurate, reproducible, high throughput and objective quantification is paramount for the study of NETs. Unfortunately, quantification is still plagued by a number of issues, such as sampling bias, insufficient objectivity, low throughput, being tedious, labor-intensive, high in cost and difficult to compare across laboratories (21–25). Luckily, recent advancements in technology allow for computational methodologies to circumvent a number of these disadvantages; being either semi or fully automated, with fully automated methods being more advantageous (25) i.e., higher in throughput, lower in cost, more sensitive and more reproducible across laboratories.

For this review we discuss the different methods for NET sample preparation followed by various computational solutions available for NET quantification. These solutions are only applicable for samples prepared for *in vitro* and *in situ* quantification of NETs. *In vivo* detection and quantification of NETs is important and it must be noted that quantification is usually done using *in situ* methods. NETs can also be detected and quantified *in vivo* by analysing serum or plasma for specific NET markers (12, 15, 26–29), however, since these do not involve computational methodologies for more automated quantification, they are not discussed in this review.

## *In vitro* and *in situ* Sample Preparation for Automated Quantification of NETs

All available techniques used to visualize NETs for quantification have comprehensibly been reviewed by de Buhr and Köckritz-Blickwede (30). Table 1 provides a complete overview of these methods including their advantages and disadvantages. Methods include SYTOX/PicoGreen (fluorescence reader or fluorescence microscopy) (1, 31, 32, 40, 41), immunolabelling (immunofluorescence microscopy [IFM] (22, 31–39), microscopy imaging flow cytometry [MIFC] (21), flow cytometry [FACS] (42), and electron microscopy (SEM and TEM) (31, 43). The most widely published and accepted techniques are SYTOX and IFM (24, 30) and thus, are the easiest to implement and with the best quantitative computational methodologies available.

**Table 1:** Summary of the main NET visualization techniques used for quantification of NETs and their advantages or disadvantages.

**Dye**	**Technique**	**Parameter**	**Advantages**	**Disadvantages**	**Selected references**
SYTOX dye/PicoGreen	FM, eye	Percentage of NET formation	Visible differentiation between necrosis and NETosis	Occasionally biased by selection of field of view, staining of DNA in NETs by DNA-intercalating dye can be blocked by cationic peptides	(1, 31, 32)
Antibody against histone-DNA complexes + Dapi	IFM, eye	Percentage of NET formation	Visible differentiation between necrosis and NETosis	Occasionally biased by selection of field of view	(31–36)
Antibody against elastase and histone-DNA complexes + Hoechst 33342	IFM, Image J	Percentage of NET formation	Unbiased software-based quantification	Clump of NETs derived from multiple cells count as one single event, occasionally biased by selection of field of view	(37)
Antibody against histone-DNA complexes + Dapi	IFM, Image J	Level of NET degradation	Unbiased software-based quantification	Occasionally biased by selection of field of view	(38, 39)
Antibody against histone-DNA complexes + Dapi	IFM, open source software	Level of NET degradation	Unbiased software-based quantification	Occasionally biased by selection of field of view	(22)
SYTOX dye/PicoGreen	FR	DNA release (μg/mL)	Unbiased	No differentiation between necrosis and NETosis, staining of DNA in NETs by DNA-intercalating dye can be blocked by cationic peptides	(31, 40, 41)
PicoGreen after nuclease digestion	FR	DNA release (μg/mL)	Unbiased	Staining of DNA in NETs by DNA-intercalating dye can be blocked by cationic peptides, less sensitive compared to antibody-mediated detection of NETs	(31, 36)
Antibody against MPO + Hoechst	MIFC	Percentage of NET formation	Unbiased, automated, enables differentiation between suicidal NETosis and vital NETosis	Imaging of cells currently undergoing NETosis and thus this method may miss those that have already lysed	(21)
Antibody against H3cit + MPO	Flow cytometry	Percentage of NET formation	Unbiased, automated, can be combined with sorting	Does not detect H3cit-independent events	(42)
Uranyl-acetate, osmium tetroxide, ruthenium red-osmium tetroxide, Cuprolinic Blue	TEM	Morphology of NET-releasing cells	Visible differentiation between necrosis and NETosis, can be used in combination with immunostaining of certain structures in NETs	Occasionally biased by selection of field of view	(31, 43, 44)
Osmium tetroxide/gold	SEM	Amount and structure of NETs-releasing cells	Visible differentiation between necrosis and NETosis, can be used in combination with immunostaining of certain structures in NETs	Occasionally biased by selection of field of view	(31, 43, 44)

*Adopted from de Buhr and Köckritz-Blickwede (30). IFM, immunofluorescence microscopy; FM, fluorescence microscopy; FR, fluorescence reader; MIFC, microscopy imaging flow cytometry; MPO, myeloperoxidase; TEM, transmission electron microscopy; SEM, scanning electron microscopy; H3cit, histone citrullination*.

SYTOX does not pass through intact cell membranes and detects NETs by staining extracellular DNA (51, 52). Its use has a number of advantages i.e., low cost and easy implementation. However, a major disadvantage is the susceptibility to false positives due to apoptosis or necrosis of neutrophils (24, 30, 53). Thus, quantification of NETs by SYTOX should always be supplemented with IFM i.e., specific labelling for NET markers, such as MPO and H3Cit (24, 30, 53). This is standard practice for *in vitro* detection of NETs and for most computational methodologies developed for these techniques.

FACS and MIFC (immunolabelling for MPO, NE, or H3Cit) also allow for robust, rapid, specific and sensitive detection of NETs in suspension (21, 30, 42). However, detection of neutrophils that have already undergone NETosis is not possible and thus cannot completely replace IFM (30). In addition, MIFC has an advantage over FACS since the technique combines FACS data as well as imaging for single cells (21, 30). Both FACS and MIFC are more challenging to implement compared to SYTOX and IFM based methods, because they are slightly more technical in nature.

As pointed out by de Buhr and Köckritz-Blickwede (30), an important consideration is the detection of NETs in *in vivo* tissue sections i.e., *in situ* detection. Since NETs are mainly quantified *in vitro* using neutrophils from peripheral blood, or ELISA based methods using serum (12, 15, 26–29), detection of NETs in localised tissue holds great importance, as was determined in placenta (7), intestine (1), kidney (27), lung (48), intracoronary material (49), and skin (50). It is possible in certain conditions that NETosis might be completely missed if not investigated *in situ*. Immunolabelling for NET specific markers on tissue sections is well-published and automated methods for their detection exist.

No automated methods for detection of NETs using SEM and TEM are available to our knowledge.

## Semi and Fully-Automated Computational Methods

Table 2 compares the advantages and disadvantages of all the computational metholdogies discussed in this review for easy comparison.

**Table 2:** Advantages and disadvantages of the main computational methodologies available to quantify NETs *in vitro* and *in situ*.

**NET staining technique**	**Compatible quantification method**	**Advantages**	**Disadvantages**	**Selected references**
SYTOX	DANA	Easy to follow tutorials, individual cell analysis, exclusion of false positives, high reproducibility and robustness, reduced analysis time	Human optimisation required, confirmation with additional NET markers required	(23, 45)
	3D-CSLM	Highly sensitive, robust	Skilled 3D-CSLM operator required, false positives, confirmation with additional NET markers required	(46)
	Plate assay	Fully automated, high-throughput, robust	False positives, non-visualization of NETs, confirmation with additional NET markers required	(24, 30)
IFM	ImageJ	Use of freeware, robust	Possible reproducibility problems across laboratories, possible sampling bias, difficult to implement, human input required, clumping cells quantified as one	(37)
	NETQUANT	Fully automated, easy to implement, reproducible and robust, individual cell analysis with multiple NET criteria, exclusion of false positives, high-throughput, advanced post-analysis data	MATLAB licence required	(25)
	Machine learning	Fully automated, high-throughput, sensitive, reproducible, exclusion of false positives	Informatics knowledge required, training for new conditions required, clumping cells quantified as one	(22, 47)
MIFC	Machine learning	Fully automated, high-throughput, sensitive, reproducible, exclusion of false positives	Informatics knowledge required, training for new conditions required	(46)
*In situ* sections	Machine learning	Fully automated, high-throughput, sensitive, reproducible, exclusion of false positives	Informatics knowledge required, training for new conditions required	(48)
	CSLM	Specific, easier to implement than machine learning protocols	Specific software required	(49)
	ImageJ	Use of freeware, robust	Additional NET markers required, subject to false positives	(50)

### Computational Methodologies Available for SYTOX Stained NETs

Two methods for semi-automated quantification for NETs stained with SYTOX exist i.e., DNA Area NETosis Analysis (DANA) (23) and another using 3-dimensional confocal scanning laser microscopy (3D-CSLM) (46). DANA involves the use of a fluorescence microscope, ImageJ macros and a Java based programme with a batch processing option. Easy to follow YouTube tutorials for DANA also exist (45). Quantifying NETs by 3D-CSLM requires skilled confocal operators. No easy to follow protocols for quantification using ImageJ exist, which could make it more difficult to implement.

For 3D-CSLM, NETs are quantified based on SYTOX green area corrected to PKH26 area (binds to membranes indicating neutrophils). Using this approach, Kraaij et al. (46) successfully detected NETs in neutrophils exposed to RA and SLE serum using 3D-CSLM. Immune complexes produce lower and more subtle NETs (54) and 3D-CSLM together with ImageJ were successful in their quantification, making it a highly sensitive semi-automated technique (46). For DANA, NET-like structures are quantified on a per cell, per image and per sample basis. DANA can also sufficiently exclude overlapping cells and fragments, which might be recognized as false positives (23). These characteristics of DANA are not possible using 3D-CSLM and ImageJ. Rebernick et al. (23) were also successful in detecting spontaneous NETs in RA neutrophils using DANA.

Rebernick et al. (23) went further to show that DANA detected a similar amount of NETs compared between to two individual readers and reduced the time for analysis from 7–10 to 1.5 h. The authors were also able to detect NETs in DAPI stained murine cells, indicating robustness for the program.

Since only SYTOX is used, time required for pipetting is significantly reduced. However, in order to confirm results from the assay, IFM of specific NET markers is likely needed (24, 30, 53). Both methods do provide unintentional bias between sample quantification, and eliminate inter-individual variability. For DANA, reproducibility of results across laboratories is also likely achievable due to its robust nature. It must be noted that in our experience, DANA still requires a large amount of human input for optimization of the program and large datasets with many different individual donors can still be time-consuming to analyse.

A more fully automated and high-throughput way to quantify NETs involves quantification of extracellular DNA using SYTOX green in a plate assay. However, this technique is known for being susceptible to false positives (24, 30) since NETs are not quantified based on morphology, but rather RFU. Even though this method is considered to be unbiased, non-visualization of NETs and non-specific staining of DNA prevents differentiation of necrosis and NETosis, and blocking of staining can occur due to the presence of cationic peptides (30).

### Computational Methods Available for IFM

For NET quantification using IFM, one semi-automated method (37) and two fully automated methods exist (22, 25). For the semi-automated method, NETs are quantified based on morphological and spatial distribution using ImageJ (37). Fully automated methods for NET quantification include using a supervised machine-learning algorithm (regression model) trained on visually annotated images (22) or NETQUANT, a MATLAB application that quantifies NETs based on a number of criteria i.e., increases in cell surface area of single cells, deformation of DNA circularity, increase in DNA:NET bound protein ratio (25).

In our experience, NETQUANT is the most user friendly and easiest to implement with the user interface being extremely easy to use (25). The machine-learning method of Coelho et al. (22) is more technically challenging since knowledge in Python is required, even though a guide on GitHub exists (47). Furthermore, since the algorithm was trained using PMA stimulated neutrophils, new training would be required for new conditions to be investigated since NETs differ by stimuli (55), whereas for NETQUANT, metadata from images is used allowing the app to adapt to different conditions and thus be really robust. The semi-automated method requires multiple steps involving ImageJ, such as segmentation, thresholding, and particle analysis to quantify NETs, making it more difficult to implement compared to NETQUANT. These additional steps could also risk sampling bias or reduce reproducibility across laboratories.

Another advantage of NETQUANT is the inclusion of the watershed algorithm (56). This allows the app to differentiate NETs in contact with each other, a feature not available in other methods. Other methods would segment clumps of neutrophils or NETs as one and not individually. The batch processing option of NETQUANT also allows for image analysis of large datasets within minutes, providing detailed single-cell data and thus allowing for more advanced post-analysis of NET formation.

All methods were successful in NET detection in varying conditions, such as PMA stimulation, cytokine induction and even in the presence of pathogens. Coelho et al. (22) and Mohanty et al. (25) went one step further and showed that their methodologies correlated well to the detection of NETs comparing two individual experts.

Currently, NETQUANT appears to be the most unbiased and uses the most stringent, biologically relevant NET definition criteria that can be applied rapidly over many different datasets.

### Computational Methods for MIFC

Apart from using the software provided for MIFC (IDEAS, considered to be semi-automatic, with batch processing possible) (21), only one fully automated methodology for NET quantification using MIFC data exists (48). The method developed by Ginley et al. (48) is a supervised machine learning algorithm for NET detection (chromatin staining only) using MIFC data. With a support vector machine (SVM), it provided a more well-rounded performance than an alternative convolutional neural network (CNN) approach. This was due to the amount of training data required. Since the algorithm only considered cells stimulated with PMA, additional training for different conditions would be necessary. Moreover, similar to Coelho et al. (22), the technical nature of the protocol can make it difficult to implement for persons lacking knowledge in informatics.

### Computational Methods Available for *in situ* Prepared Sections

The same authors as above (48) used an unsupervised learning method on confocal images obtained from thin sections of lung tissue in a murine fungal pneumonia model stained for DNA, MPO and histone H1. The percent pixels of H1, present in decondensed nuclei colocalised with MPO, was the classification criteria. Applying deep CNN to this co-localisation data, a supervised approach can be applied. The pixel wise sensitivity/specificity was 0.99/0.98 for NET detection on 14 images using the unsupervised learning method. Their supervised CNN method uses object patches that had an object-wise holdout sensitivity/specificity of 0.86/0.90 on 631 object patches (from two images). This is the most automated method for NET detection in tissue sections. Unfortunately, as with other machine-learning methodologies, it can be challenging to implement.

Santos et al. (49) developed a semi-automated method for NET detection in paraffin-embedded intracoronary thrombus aspirate samples. Using confocal microscopy, NETs in the sections are detected by staining for DNA, MPO, and H3Cit. Thus, the method is highly specific and easier to implement than that of machine-learning algorithms proposed by Ginley et al. (48). Naturally, analysis is slightly more tedious and slower than the fully automated methods of Ginley et al. (48). A disadvantage is the requirement for specific analysis software i.e., SF SOFTWARE VERSION 2.6.07266 (LEICA). Since the method is largely based on co-localisation, development of methods using Imaris might provide more robust methods for cross-laboratory application.

NETs were also generated *in vivo* using a *Mycobacterium tuberculosis* guinea pig model and quantified *in situ* using semi-automated methods (50). Using ImageJ, the authors quantify NETs based on pixel density per area. Tissue sections were stained using Hoechst. Thus, NET quantification was based on an increase in the observed DNA area. As mentioned, this is not specific to NET formation which requires additional staining for NET markers, such as MPO, elastase etc. Thus, the authors went further to prove that the increase in DNA area is colocalised with certain NET markers. A more accurate method involving the quantification of NETs based on specific markers, such as MPO would prove to be more accurate i.e., that of Santos et al. (49).

## Conclusion

Imaging of NETs can be a tedious task subject to sampling bias. Fortunately, a large number of groups are working towards high quality and easy to implement software packages that allow for high throughput and accurate quantification of NETs. This further will allow for reduction in sampling bias and allow for better reproducibility across laboratories.

## Author Contributions

SvB conceptualized and wrote the manuscript. LV prepared the table. CN assisted with references. SR, SH, and PH read and revised the manuscript.

### Conflict of Interest Statement

The authors declare that this study received partial funding from Celgene, USA. The funder had no role in the design of the mini review, decision to publish or preparation of the manuscript. The authors declare that the research was conducted in the absence of any commercial or financial relationships that could be construed as a potential conflict of interest.
